# Recent Advantages on Waste Management in Hydrogen Industry

**DOI:** 10.3390/polym14224992

**Published:** 2022-11-18

**Authors:** Alexander V. Shchegolkov, Aleksei V. Shchegolkov, Natalia V. Zemtsova, Yaroslav M. Stanishevskiy, Alexandre A. Vetcher

**Affiliations:** 1 Institute of Technology of the Department of Technology and Methods of Nanoproducts Manufacturing, Tambov State Technical University, 392000 Tambov, Russia; 2 Department of Chemical Technology, Platov South-Russian State Polytechnic University (NPI), 132 Enlightenment Str., 346428 Novocherkassk, Rostov Region, Russia; 3 Department “Technique and Technology for Obtaining Nanoproducts”, Tambov State Technical University, 106 Sovetskaya Str., 392000 Tambov, Russia; 4 Institute of Biochemical Technology and Nanotechnology (IBTN), Peoples’ Friendship University of Russia (RUDN), 6 Miklukho-Maklaya Str., 117198 Moscow, Russia; 5 Complementary and Integrative Health Clinic of Dr. Shishonin, 5 Yasnogorskaya Str., 117588 Moscow, Russia

**Keywords:** hydrogen storage (tank), nanocomposite(s), nanotubes, waste management

## Abstract

The turn to hydrogen as an energy source is a fundamentally important task facing the global energetics, aviation and automotive industries. This step would reduce the negative man-made impact on the environment on the one hand, and provide previously inaccessible power modes and increased resources for technical systems, predetermining the development of an absolutely new life cycle for important areas of technology, on the other. The most important aspect in this case is the development of next-generation technologies for hydrogen industry waste management that will definitely reduce the negative impact of technology on the environment. We consider the approaches and methods related to new technologies in the area of hydrogen storage (HS), which requires the use of specialized equipment equipped with efficient and controlled temperature control systems, as well as the involvement of innovative materials that allow HS in solid form. Technologies for controlling hydrogen production and storage systems are of great importance, and can be implemented using neural networks, making it possible to significantly improve all technological stages according to the criteria of energy efficiency reliability, safety, and eco-friendliness. The recent advantages in these directions are also reviewed.

## 1. Introduction

Enhancement of environmental friendliness at all levels of energy resources employment is still a priority task that can be solved by considering hydrogen as an energy source [1]. Hydrogen is well-known as a carbon-free energy source, and its properties have been exhaustively studied by generations of scientists. Its wide application could potentially replace hydrocarbons and, accordingly, emissions of gaseous carbon in a variety of forms.

The application of hydrogen is based on the employment of fuel cells, which are efficient energy converters with significant potential for use in transport and other areas of energy production [2]. Fuel cells have an energy conversion efficiency of about 60–70%, which is significantly higher than for devices using the Carnot cycle. Currently, fuel cells have been demonstrated to be safe and efficient devices that can ensure a fast refueling process and energy efficiency [3]. Cathodic and anodic reaction—implemented on FC with a pronounced anode and cathode—is characterized by the fact that hydrogen is ionized, and its energy is released with the accumulation of electrons on the FC anode’s surface. At the same time, oxygen is reduced at the cathode, which indicates anodic oxidation and cathodic reduction [4].

It should be noted that the main material for creating fuel cells is titanium, which corrodes during operation. To reduce the intensity of the corrosion process, a cathodic deposition of tungsten trioxide on the titanium surface can be used [5].

There are several main directions for the use of hydrogen that are widespread at the moment. The most important applications of hydrogen are:

- Chemical industry—synthesis of ammonia, methanol, and hydrocarbons, as well as the recovery of metals from their oxide form [6].
- Nanotechnology—in the process of CVD synthesis for the reduction of metal oxide catalysts in the synthesis of carbon nanotubes (CNTs) [7,8]. The reaction takes place at 600–1000 °C in propane–butane flow.
- Energetics—an energy source for electric and thermal power engineering [9].
- Petrochemistry—oil refining (hydrogenation purification of petroleum products—hydrodesulfurization) [10].
- Transportation—cars running on gaseous and liquid hydrogen [11,12].

In this latter case, a distinction should be made between passenger cars [13] and commercial cargo and passenger transportation means [14,15].

The implementation of hydrogen as the main energy source in various types of buses with FC in their design features the fact that hydrogen is converted into electrical energy, and the by-product is water vapor, which condenses into water in the environment [16]. The approach presented—in which H_2_ is generated electrically to split water into O_2_ and H_2_ or by chemical conversion of methane to H_2_ (loop conversion of methane with steam on anti-coking compounds CeO_2_/La_0.9_Sr_0.1_Fe_1−x_Ni_x_O_3_) [17]—makes it possible to abandon the use of petroleum products such as motor oils. This will also have a positive and tangible impact on the ecological situation of megacities, as it will eliminate the need to recycle used engine oils. The transition to a hydrogen energy system is likely to be based on H_2_, obtained as a result of reforming natural gas or electrolysis.

The most widespread use of hydrogen is primarily in the field of motor transport, which needs environmentally friendly and affordable energy sources that are capable of replacing hydrocarbons. Another option that should be considered is the generation of energy at a thermal power plant [18], and in this case, one practice involves the partial mixing of hydrogen with methane or other gaseous fuels based on hydrocarbons [19].

The transition to hydrogen as the main type of fuel could form and transform the design of new types of internal combustion engine, and in particular, hydrogen rotary Wankel engines could find distribution [20]. Other types of vehicles in which hydrogen can be used include aircraft or air transport [21] and unmanned aerial vehicles [22]. Commercial hydrogen production currently depends mainly on steam natural gas reforming [23] and coal partial oxidation [24]. Clean production using both biomass and solar energy production methods is on its way [25].

Thus, there is a widespread practice of using hydrogen, which has the possibility of serving as a basis for an entire direction of research. For the successful dissemination of these achievements, however, several fundamentally important and significant problems need to be solved, including the safe generation and storage of hydrogen. At the same time, it should be borne in mind that polymer waste can be used as a source of cheap raw materials for producing hydrogen and related high-performance materials. There is also a fundamental possibility when using new control technologies related to artificial intelligence in the process of hydrogen synthesis and storage.

## 2. Design and Thermodynamics of HS Tank

HS is currently a “bottleneck” for the implementation of the use of hydrogen as renewable energy. A key challenge for the full development of hydrogen-based technologies is the safe, efficient, and economical storage of hydrogen.

**Figure 1:**
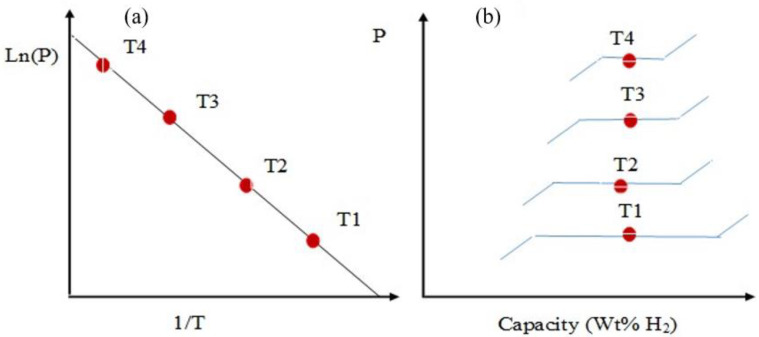
Van ’t Hoff diagram of metal hydride (MH) (**a**) and phase diagram (**b**).

Practical materials for HS should have the ability to undergo a reversible hydrogenation/dehydrogenation process, which is determined by the binding energy of hydrogen atoms. The PCT curve is a graph representing the dependence of pressure on composition at different temperatures. Figure 1 demonstrates a graphical interpretation of the Van ’t Hoff equation [26], which indicates the dependence of the logarithm of the equilibrium desorption pressure on the reciprocal temperature (T) (Figure 1a), as well as the dependence of the amount of hydrogen accumulation on pressure (Figure 1b).

It is necessary to consider the temperature regimes that are characteristic of the storage of liquid hydrogen. Transient heat transfer plays a leading role in multilayer insulation (MLI) in combination with a steam-cooled shield (SCS) used in liquid HS tanks. In [27], the profile of the transition temperature and the change in the heat flux of MLI and SCS were predicted and analyzed, which can help optimize the operating parameters for liquid HS.

Considering the technological aspects of HS in porous materials, a new design concept for a portable HS tank was identified [28]. Within the framework of this concept, a storage method is used in which low pressure and cryogenic temperature are realized. To maintain the cryogenic temperature, three-layer insulation was used, allowing for at least 12.5 days without the need for an external cooling circuit to maintain the optimum temperature. The HS tank is portable and can be used in various types of FS electric vehicles (FCEVs). As a tank filler, porous absorbents can be used, which form such storage conditions at which a temperature of 77 K and a pressure below 100 bar is maintained. The presented parameters are significantly lower than the internal pressure of 700 bar in commercial type IV tanks [29], thus improving safety and reducing the risk of explosion.

The safety of the use of HS tanks with TPRD under fire conditions can be improved by using composite materials [30]. HS tank model inputs include thermal parameters of the hydride and tank materials, fire heat flow into the tank, diameter TPRD, and initiation delay time TPRD. Non-stationary heat transfer from the environment through the tank wall and lining to hydrogen leads to decomposition of the composite resin for wrapping and melting of the lining. The lower limit of the diameter of the TPRD hole is sufficient to prevent the tank from bursting in the case of fire.

With respect to the option of storing hydrogen in liquid or solid form, storage in the solid state is preferable. This is due to the improvement in explosion and fire safety, and also provides better volumetric and gravimetric density, which improves the weight and size parameters of hydrogen accumulators. It should be noted that hydrogen in solid-state storage is bound by physicochemical forces [31]. The strength of the interaction between hydrogen and the carrier material varies from weak van der Waals interactions, which are characteristic of the physisorption binding of molecular hydrogen, to the strong chemisorption binding of atomic hydrogen [32]. The storage density of hydrogen can be improved by using hydride-type materials; hydrogen is packed with a HH distance of up to 170 kg/m^3^, which is more than twice the density of liquid hydrogen.

**Figure 2:**
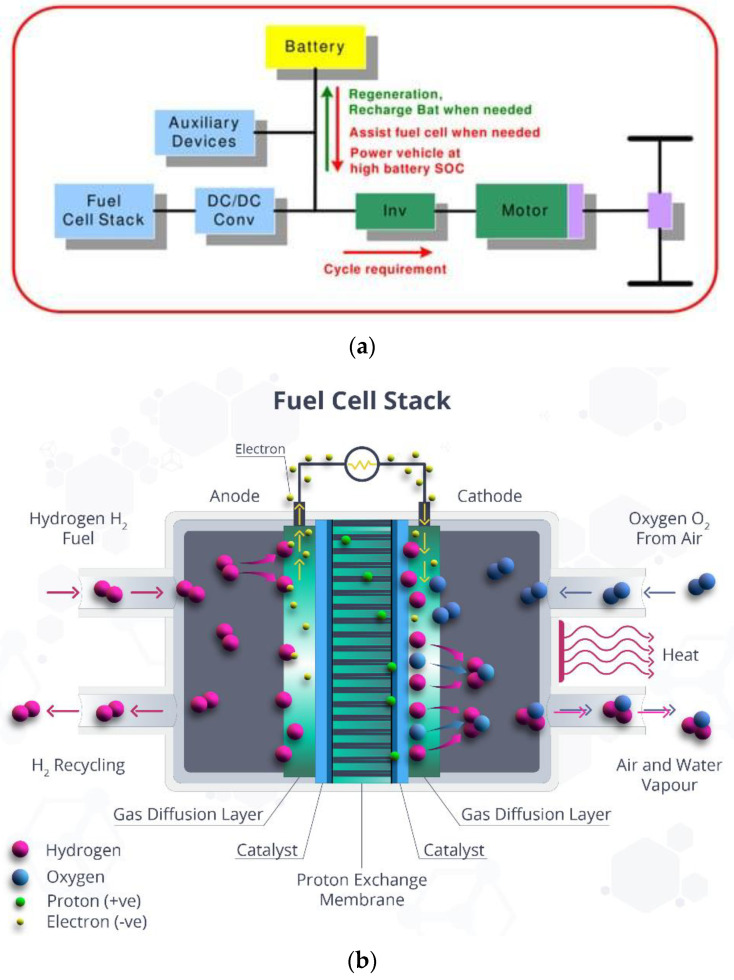
(**a**) Schematic of FC-based electric vehicle [29]. (**b**) The process of charging the cell with hydrogen.

When storing hydrogen based on MH, it is necessary to use specialized heat exchangers or thermal control systems due to both endothermic and exothermic reactions taking place during the filling and unloading of MH tanks (Figure 2). In the absence of a heat exchanger or a suitable temperature range, the operation of the MP will have a negative influence, leading to the instability of the supply of hydrogen in FS systems. In [33], the influence of the tank surface temperature on the hydrogen consumption and hydrogen flow characteristics for the HS system MH of an electric vehicle operating on hydrogen FC was studied. Various temperature values were provided with the help of an external heat circulation device and a heat exchanger inside the MH tank. The FC operated in a power range from 200 to 600 W, and was regulated depending on the temperature and flow rate of the pumped reservoir [34].

MH cartridges based on the hydride of La_0.75_ Ce_0.25_ Ni_5_ can be used for HS [35]. The low thermal conductivity of MH is a limiting factor with respect to the technological problems of HS. To improve the thermal and physical characteristics of MH, metal foam with a porosity gradient can be used [36].

## 3. Composite Materials for HS—Thermoset Composites

The safe storage of a reasonable amount of hydrogen is associated with many problems related to the method and materials. Hydrogen accumulator materials can be of different types:

1. Dissociative material in which molecular hydrogen is dissociated into hydrogen atoms occupying internodes;
2. Materials with chemically bonded hydrogen;
3. Materials that adsorb molecular hydrogen, in which molecular hydrogen attaches to the surface due to weak interactions, such as the Van der Waals force or physical sorption.

The ability of certain materials to accumulate hydrogen depends on the structure and type of interaction with hydrogen. There are some new materials for HS. Storage of hydrogen in solid form can be briefly divided into the following categories:

1. MHs;
2. Hydrides based on light metals;
3. Chemical hydrides (complex hydrides);
4. Nanostructured materials (adsorption of molecular hydrogen).

**Figure 3:**
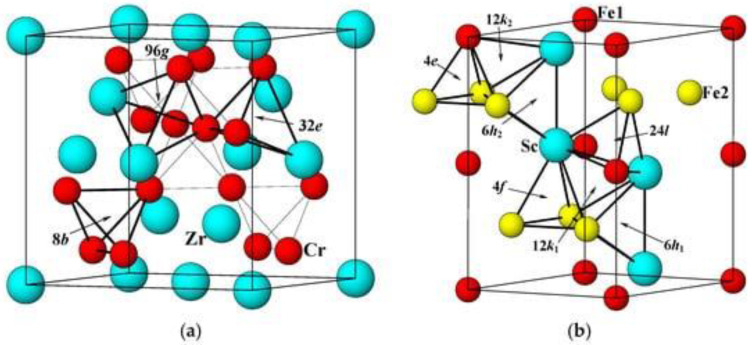
Elementary cells for the structures ZrCr_2_ (C15) (**a**) and ScFe_2_ (C14) (**b**). Various tetrahedral hydrogen filling sites (A2B2, AB3 and B4) are indicated by arrows [34].

Intermetallic compounds can be used as hydrogen accumulators [37,38]. This is due to the peculiarities of their atomic structure, in which interstices with the optimal binding energy for hydrogen are observed, forming the process of absorption or desorption under conditions close to standard. In this regard, for the storage of hydrogen, the class of compounds of the Laves phase with the formula unit (AB) needs to be taken into consideration [39]. Because they change from one to the other on heating and cooling (usually C14 at high temperatures and C15 at low temperatures), hydrogen absorption–desorption can be thought of as a phase change process. Structure C15 is an fcc structure containing six atoms per unit cell, while structures C14 and C36 are hexagonal structures containing 12 and 24 atoms per unit cell. Figure 3 demonstrates the crystal structures of C14- and C15-type alloys. Ideally, the lattice parameters are closely related in each structure and between structures. However, in real MH alloys with a predominance of C14, the c/a ratio is slightly lower than the theoretical value (223--√≅1633) [40]. Three types of position are available for tetrahedral hydrogen filling positions (A2B2, AB3 and B4) for both C14 and C15 structures, as shown in Figure 3. There are no octahedral positions at all in the Laves phases, so further discussion will focus only on tetrahedral positions [41].

High-temperature MH, such as MgH_2_, is considered one of the most promising HS technologies [42]. However, there are two main bottlenecks, including the low rate of H_2_ absorption and the low power of the MH reactor. In this regard, heat removal from the MG tank plays a crucial role in the HS process. The results show that the charging time is significantly reduced by increasing the number of air channels, as the heat transfer rate is significantly improved. When the initial coolant temperature rises, the charging time increases; however, as the Reynolds number of the coolant increases and the hydrogen inlet pressure increases, the absorption process accelerates. The recommended configuration of the heat exchanger is introduced taking into account both the loading time and production constraints. It is shown that the loading of a new multi-zone hydrogen energy storage using four air channels is approximately 30 min, which provides a more applicable hydrogen fuel system.

Ref [43] presents experimental studies concerning the absorption of H_2_ in a solid-state HS device based on LmNi_4.91_Sn_0.15_ with integrated cooling tubes (ECT). MH with ECT 36 and 60 loaded with 2.75 kg LmNi served as the basis for a hydrogen accumulator that implements various modes of supply pressure (10–35 bar), absorption temperature (20–30 °C) and coolant flow (2.2–30 L/min).

It has been found that at any given absorption temperature, the rate of H_2_ absorption and the amount of absorbed H_2_ increase when the H_2_ supply pressure rises to about 35 bar. Assuming the supply of H_2_ at a pressure of 35 bar and an absorption temperature of 30 °C, using oil as a coolant at a flow rate of 3.2 L/min, the maximum absorption of hydrogen is ≈1.2 wt.% for 10 min for 36 ECT and 8 min for 60 ECT. Under absorption conditions with a supply pressure of 25 bar, a water flow rate of 30 L/min and an absorption temperature of 30 °C, the absorption time in the reactor with 60 ECT was reduced to 5 min. Most metals are able to absorb hydrogen reversibly. Undoubtedly, the MG reactor (MR) is the main device used to achieve the desired stability and integrated operation of the HS system.

It has been found that at any given absorption temperature, the rate of hydrogen absorption and the amount of hydrogen absorbed increases as the hydrogen supply pressure rises to about 35 bar. Assuming a hydrogen supply pressure of 35 bar and an absorption temperature of 30 °C, using oil as the heat transfer medium at a flow rate of 3.2 L/min, the maximum absorbed hydrogen is ≈1.18 wt% per 10 min for 36 ECT and 8 min for 60 ECT. Under absorption conditions with a supply pressure of 25 bar, water flow 30 L/min and absorption temperature 30 °C, the absorption time in the reactor from 60 ECT is reduced to 5 min. The majority of metals can reversibly absorb hydrogen. Undoubtedly, the MH reactor (MHR) is the main device used to achieve the stable and integrated operation of HS systems desired.

Furthermore, each of the materials of this class in the form of nanocomposites will be considered to give a reasonable explanation for the improvement in the storage conditions of hydrogen as an energy source.

## 4. Hydrogen Generation and Storage on the MoS_2_-Containing Materials

It appears that MoS_2_ possesses great prospects as a cost-effective replacement for Pt for catalysis of the hydrogen evolution reaction (HER) in water, despite its claimed catalytic efficiency being lower than that of Pt. The latter is known to be the best HER catalyst, but it appears to be too expensive for mass production in the hydrogen industry. Monolayer (ML) MoS_2_ films were grown using CVD processes. Substrates are able to affect the catalytic activity of MoS_2_ in two ways: by forming an interfacial tunnel barrier with MoS_2_; and by changing the chemical nature of MoS_2_ through charge transfer (proximity doping).

The catalytic characteristics can be further improved such that they are even better than those of Pt by crumpling films on flexible substrates, since the Tafelian slope of the film is significantly reduced in the presence of compression deformation caused by crumpling [44]. MoS_2_ can be used for the hydrogen evolution reaction (HER). Thermal effects formed an effective electron transfer in the atomic MS MoS_2_ and at the electrolyte–catalyst interface, leading to an increase in the activity of GWR [45].

Vertically grown MoS_2_ nanolists supported by conductive carbon nanotubes and reduced graphene oxide (CNT-rGO) on traditional Vietnamese paper (MoS_x_/CNT-rGO/VTP) can be used for the electrochemical reaction of hydrogen evolution (HER). The catalyst demonstrates excellent electrocatalytic activity of HER, including a low initial potential of 190 mV, a Taffel slope of 59 mV/°, and excellent stability in an acidic electrolyte solution [46].

The Taffel slope shows the dependence of the catalytic reaction rate on the applied overvoltage. The smaller the Taffel slope, the faster the reaction rate increases when applying overvoltage. Typically, the Tafel slope (n) of an electrochemical reaction is dictated by the rate-determining reaction stage, and can be written as a function of the number of electrons involved (z) and the charge transfer coefficient (α) of the limiting factor. The extraction step is n = 2.3zRT/aF, where R is the ideal gas constant, T is the absolute temperature, F is the Faraday constant [47]. For MoS2 materials, which are usually found in an acidic environment, three reaction stages can be involved:

- Primary discharge stage (Vollmer reaction):

H_3_O^+^ + e^−^ → Hads + H_2_O

- Electrochemical desorption stage (Geyrovsky reaction):

Hads + H_3_O^+^ + e^−^ → H_2_ + H_2_O

- Recombination stage (Tafel reaction):

Hads + Hads → H_2_

The edge regions of MoS_2_ are catalytically active in hydrogen evolution reactions (HER). F atoms with high electronegativity are doped into the edge nodes of MoS_2_, leading to a fivefold increase in activity compared to the initial edges, which is explained by the more moderate binding energy for hydrogen particles [48].

The photocatalyst of the MoS_2_@ MoO_3_ heterojunction with the (S)-scheme stage was prepared by partial sulfidation in situ. The excellent design of the MoS_2_@ MoO_3_ interface nanomaterials provides a high rate of surface reaction of hydrogen evolution. The on-site vulcanization strategy gradually causes corrosion from the outside to the inside. The rate of hydrogen formation is 12,416.8 mmol/h·g [49].

The improvement of MoS_2_ properties can be achieved by the formation of a chemical bond between the MoS_2_ nanolayer on graphene and vacancies. There is a clear decrease in the metallic state of the MoS_2_ nanolayer as electrons are transferred to form a strong contact with the restored graphene substrate. The absence of a metallic state associated with unsaturated atoms in the peripheral regions in turn changes the activity of hydrogen release. The easiest path of evolution is from the marginal regions of the Mo, and the presence of graphene leads to a decrease in the energy barrier from 0.17 to 0.11 eV. The evolution of H_2_ from the S-edge is complicated due to an increase in the energy barrier from 0.43 to 0.84 eV [50].

The formation of S-vacancies on the basic plane of MoS_2_ by electrochemical desulfurization makes it possible to improve the process of hydrogen generation. The formation of S-vacancies is possible on various 2H- MoS_2_ nanostructures. By changing the applied desulfurization potential, it is possible to vary the degree of desulfurization and the resulting hydrogen release activity [51].

A three-dimensional hierarchical hybrid composite of MoS_2_, reduced graphene oxide (GO) and CNT demonstrates excellent electrocatalytic activity and stability in the hydrogen evolution reaction, with a low initial potential of only 35 mV, a Taffel slope of ~38 mV/°, and an apparent exchange current density of 74.25 mA/cm^2^. The excellent hydrogen release activity is due to the synergistic effect of MoS_2_ with its electrocatalytically active edge regions and excellent electrical coupling with the underlying graphene and CNT grid [52].

Hierarchical MoP- MoS_2_ electrocatalysts on hollow carbon spheres co-doped with N, P and S (MoP- MoS_2_/HCS) demonstrate remarkable HER characteristics. MoP- MoS_2_/HCS not only exhibit significant electrocatalytic activity at low overvoltages (71 mV and 125 mV in 1.0 M KOH and 0.5 M H_2_SO_4_, respectively) at a current density of 10 mA/cm^2^, they also exhibit outstanding stability with respect to its process [53,54].

## 5. Processing of Composite Materials from the Hydrogen Industry

Global industry actively uses various types of plastic, which inevitably leads to the formation of a large amount of plastic waste. More than 60% of used plastic ends up in landfills or is incinerated, which harms the environment and the ecosystem as a whole [55]. Thermal processing by pyrolysis and gasification of plastic waste into fuel and chemical products has been identified as a promising technology for solving the problems of plastic waste [56]. Pyrolysis is a method of thermochemical treatment of plastic waste, which can solve such pollution problems, as well as restore valuable energy and products such as oil and gas. Pyrolysis of solid plastic waste has become important because it offers great advantages in terms of environmental pollution and reducing the carbon footprint of plastic products by minimizing carbon monoxide and carbon dioxide emissions compared to combustion and gasification [57].

In [58], a simple and highly efficient method initiated by microwave plasma discharge for the decomposition of plastics into hydrogen and carbon nanotubes was proposed. Iron-based catalysts applied to activated carbon calcined at 400 °C showed the best catalytic activity due to excellent physicochemical properties. H2 was rapidly released in 25 s, with a hydrogen efficiency of more than 85%.

In [59], pyrolysis and catalytic decomposition of polypropylene were carried out in the technological process for the production of hydrogen and carbon nanomaterials. A series of new Fe/Ni catalysts was prepared, and the effect of the active metal component of the catalyst (Fe, Ni, FeNi) and the synthesis method (sol–gel and impregnation) was studied. The results showed that the production of hydrogen and solid matter occurred in descending order with loading of Fe-Ni, Fe and Ni, while the catalysts prepared by sol–gel were more catalytic than their impregnated counterparts. FeNi (SG) demonstrated optimal activity in the production of 25.14 mmol/g of hydrogen plastic and 360 mg/g of high-quality plastic made of carbon nanomaterials.

**Figure 4:**
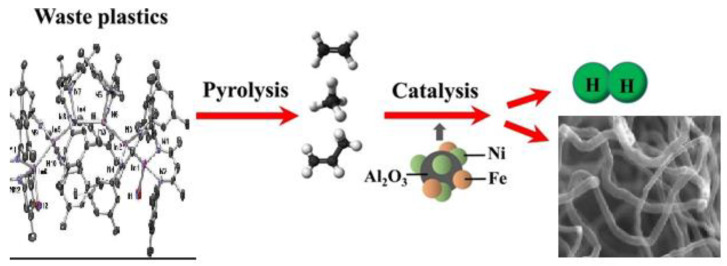
Schematic of pyrolysis of polymer with catalyst and hydrogen release.

In [60], the use of Ni-Fe catalysts was studied for the catalytic pyrolysis of plastic waste to produce hydrogen and CNT, as well as the influence of the composition of the catalyst and carrier materials. The bimetallic Ni-Fe catalyst showed higher catalytic activity in H_2_ yield than monometallic Ni or Fe catalysts due to the optimal interaction between the metal and the carrier. The effect of steam supply and catalyst temperature on the yield of CNT (287 mg/g of plastic) and hydrogen (31.8 mmol H_2_/g of plastic) is optimal at 800 °C in the presence of a bimetallic Ni-Fe/γ-Al_2_O_3_ catalyst (Figure 4).

**Figure 5:**
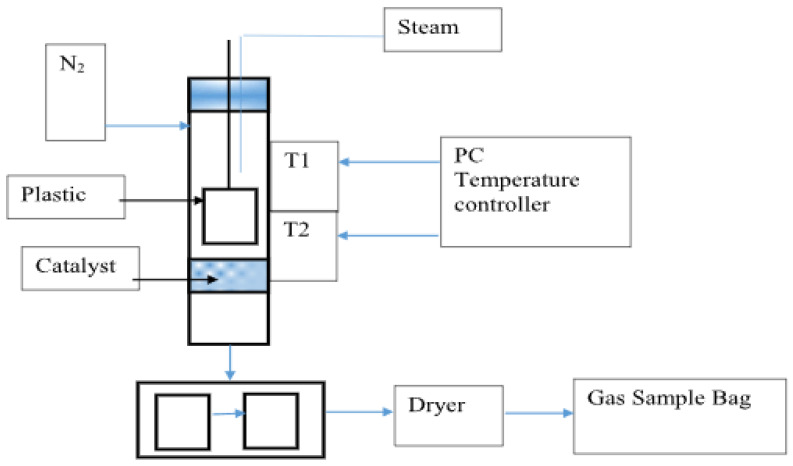
Schematic diagram of the pyrolysis–catalysis process of plastic waste.

For the catalytic pyrolysis of plastic waste, a two-stage fixed-bed reactor (with pyrolysis zone, height 310 mm; bottom: pyrolysis zone, height 310 mm) was reported (Figure 5 [60]). Three series of experiments were conducted to determine the technological parameters for the generation of hydrogen and CNTs: with the use of Fe/γ-Al_2_O_3_, Fe/α-Al_2_O_3_, Ni/γ-Al_2_O_3_, Ni/α-Al_2_O_3_, and Ni-Fe/γ-Al_2_O_3_ catalysts; with ratios of mass of steam to mass of plastic of 0, 0.3, 1, and 2.6; and with catalytic temperatures of 700, 800, and 900 °C.

The analysis of waste gases and thermodynamic calculations [61] showed that the H_2_ emission, through the decomposition of the by-product CH_4_, acted as a thermal micronization medium, where Fe_x_O_y_ is gradually restored after the waste is converted into activated carbon (CA). The resulting CA is then effectively involved in the catalytic decomposition of H_2_O, leading to the microgeneration of secondary H_2_, creating a controllable system. Thus, the fast release of H_2_ from the system was eliminated, resulting in improved recovery of Fe_x_O_y_ due to a simplified H_2_ microgeneration/regeneration process.

A two-stage catalytic pyrolysis steam reforming process with MSM-41 mesoporous Fe-Ni bimetallic catalysts was used to produce hydrogen-enriched synthesis gas from a simulated mixture of waste plastics (SMWP) [62]. Various weight ratios of Fe:Ni catalyst materials (0:20, 5:15, 10:10, 15:5, 20:0) were investigated to determine the effect on H_2_ production. The results showed that the combined presence of Fe and Ni leads to a synergistic increase in the total gas yield and the formation of hydrogen and carbon monoxide. The highest gas yield of 95 wt.%, the highest H_2_ yield of 46.1 mmol H_2_/g plastic, and the highest CO yield at 31.8 mm/g plastic are characteristic of the Fe/Ni/MCM-41 catalyst (1:1). This catalyst gives a hydrogen yield of 46.7 vol.% and a CO yield of 32.2 vol.% [62].

Ni/SiO_2_ and Fe/SiO_2_ catalysts with metal particles of different sizes were studied in the production of hydrogen and CNTs during the catalytic processing of polypropylene waste using a two-stage fixed-bed reaction system we reported [63]. The results show that Fe-based catalysts, in particular those with large particle size (~80 nm), gave the highest hydrogen yield (~25.60 mmol H_2_/g of plastic) and the highest carbon yield (29 wt. %), as well as the largest proportion of graphitic carbons (according to the analysis of the TPO of the reacted catalyst).

In the process of hydrogen production, a more complex three-component catalyst can be used [64]. The yield of hydrogen increased with an increase in the gasification temperature from 600 to 900 °C for both Ni-Mg-Al and industrial nickel catalysts. The maximum hydrogen production was 52% of the maximum theoretical amount of hydrogen available in polypropylene, which is 22–38 g H_2_/100 g of polypropylene obtained with a Ni-Mg-Al catalyst, at a gasification temperature of 800 °C and a water flow rate with an injection speed of 28–46 g/h.

Real waste plastics contain dissimilar materials. In [64], the production of H_2_ from pyrolysis–catalytic steam reforming of polyethylene, polystyrene (PS), and polyethylene terephthalate waste plastics was considered. The highest yield of hydrogen (125 mmol/g plastic) was obtained with PS at a catalyst temperature of 900 °C and a steam hourly space velocity of 7.59 g/(h/g catalyst) with 10 wt% Ni/Al_2_O_3_) catalyst. The high catalyst temperature (900 °C) and the optimized steam rate significantly increase the hydrogen yield. The authors found that Ni/Al_2_O_3_ has the highest selectivity and catalytic activity for hydrogen yield.

Comparison of energy spendings of various methods is presented in Table 1, from which it can be observed that that with the highest energy consumption is water electrolysis.

## 6. Conclusions

The use of hydrogen includes a variety of directions from chemical technologies to unmanned aerial vehicles. The field of motor transport is a key one for the mass development of water generation and storage technologies.

New technologies in the field of HS are based on the use of specialized equipment with temperature control systems, as well as the use of innovative materials that make it possible to store hydrogen in solid form. For the storage of hydrogen in solid form, Mg- or Li-based MHs in the form of classical composites or having nanostructured morphology show the greatest efficiency. Hydrides of nanometals can be obtained using the “bottom-up” and “top-down” strategies. It should be noted the principal possibility of using carbon materials for HS, namely carbon nanotubes, both single-layer and multi-layer. To explain the mechanisms of the catalytic effect of impurities in the metallic alloy on the properties of CNTs with respect to hydrogen accumulation, a spillover mechanism or hydrogenation of the Cubas type can be used.

MoS_2_ and the wide variety of composites based on it exhibit serious prospects in technologies for the production and storage of hydrogen. It is obvious that further technological development will provide novel solutions.

To produce hydrogen, waste polymer products are used—the processing of which is realized on the basis of catalytic pyrolysis. Catalytic pyrolysis also makes it possible to obtain carbon nanotubes that can be used for HS. Control technologies for hydrogen production and storage systems are implemented on the basis of neural networks, making it possible to significantly improve all technological stages according to the criteria of energy efficiency and reliability, as well as safety. An analysis of the energy costs for hydrogen production shows that direct current electrolysis is the most expensive, and thermal decomposition (pyrolysis) is less expensive.

It is worth underlining that the contemporary situation in the energy market does not demonstrate logical tendencies and therefore does not allow us to make clear predictions on the directions of hydrogen industry development in the closest future. However, the long-term prospective requires continued scientific research in this direction [65,66,67].

## Figures and Tables

**Table 1:** Comparison of energy efficacy of different processes of hydrogen generation.

Process of Hydrogen Generation	Specific Heat Consumption for Endothermic Reactions, *q_x_* (kJ/kg H_2_)	Specific Consumption of Reference Fuel (rf) to Provide Endothermic Reactions, *b* (kg rf/kgH_2_)	Specific rf Consumption for Production of kg H_2_, b* (kg rf/kgH_2_)	The Ratio of the Calorific Value of the Total Amount of Fuel to H_2_ on kg H_2_ Δ (kJ/kJ)
Conversion of Methane with Steam in Reactors with a Fluidized Bed of a Dispersed Catalyst CH_4_ + 2H_2_O→ CO_2_ + 4H_2_ (by-product CO_2_)	34,987	1.32	4.74	1.12
Carbon gasification of solid fuel with water vapor C + 2H_2_O→ CO_2_ + 2H_2_ (by-product CO_2_)	67,958	2.89	6.24	1.37
CH_4_ pyrolysis at 1350 °C CH_4_→2H_2_ + C (by-product C)	18,922	0.72	7.56	1.815
Water electrolysis 2H_2_O→ H_2_ + 0.5O_2_ + H_2_O (by-product O_2_)	214,268.4	27.77	27.77	1.77

## Data Availability

The data presented in this study are available on request from the corresponding author.

## References

[1] Yang Y., Tong L., Yin S., Liu Y., Wang L., Qiu Y., Ding Y. (2022). Status and challenges of applications and industry chain technologies of hydrogen in the context of carbon neutrality. J. Clean. Prod..

[2] Nimir W., Al-Othman A., Tawalbeh M., Al Makky A., Ali A., Karimi-Maleh H., Karimi F., Karaman C. (2021). Approaches towards the development of heteropolyacid-based high temperature membranes for PEM fuel cells. Int. J. Hydrogen Energy.

[3] Modarres M., Kaminskiy M., Krivtsov V.V. (2017). Reliability Engineering and Risk Analysis: A Practical Guide.

[4] Olabi A.G., Abdelkareem M.A., Wilberforce T., Sayed E.T. (2021). Application of graphene in energy storage device—A review. Renew. Sustain. Energy Rev..

[5] Shchegolkov A.V., Lipkin M.S., Shchegolkov A.V. (2022). Preparation of WO_3_ Films on Titanium and Graphite Foil for Fuel Cell and Supercapacitor Applications by Electrochemical (Cathodic) Deposition Method. Russ. J. Gen. Chem..

[6] Hu Y., Xue N., Zhang Y., Hu P. (2022). An eco-friendly vanadium precipitation method through solution-phase hydrogen reduction with nickel catalysis. J. Taiwan Inst. Chem. Eng..

[7] Machado G., Coelho C. (2021). Vertically-aligned carbon nanotube at low pressure by cold-wall thermal CVD using a two-phase deposition step. Carbon Trends.

[8] Chan K., Maznam N., Hazan M., Ahmad R., Sa’Ari A., Azman N., Mamat M., Rahman M., Tanemura M., Yaakob Y. (2022). Multi-walled carbon nanotubes growth by chemical vapour deposition: Effect of precursor flowing path and catalyst size. Carbon Trends.

[9] Rejeb O., Alirahmi S.M., Assareh E., Assad M.E.H., Jemni A., Bettayeb M., Ghenai C. (2022). Innovative integrated solar powered polygeneration system for green Hydrogen, Oxygen, electricity and heat production. Energy Convers. Manag..

[10] Speight J.G. (2014). Effects in Refining. High Acid Crudes.

[11] Jafari H., Safarzadeh S., Azad-Farsani E. (2022). Effects of governmental policies on energy-efficiency improvement of hydrogen fuel cell cars: A game-theoretic approach. Energy.

[12] Ku A.Y., Reddi K., Elgowainy A., McRobie J., Li J. (2022). Liquid pump-enabled hydrogen refueling system for medium and heavy duty fuel cell vehicles: Station design and technoeconomic assessment. Int. J. Hydrogen Energy.

[13] Boretti A. (2010). Comparison of fuel economies of high efficiency diesel and hydrogen engines powering a compact car with a flywheel based kinetic energy recovery systems. Int. J. Hydrogen Energy.

[14] Coleman D., Kopp M., Wagner T., Scheppat B. (2020). The value chain of green hydrogen for fuel cell buses—A case study for the Rhine-Main area in Germany. Int. J. Hydrogen Energy.

[15] Charters D. (2016). A comparison of energy vectors in powering hybrid buses. Renew. Energy Focus.

[16] Emonts B., Schiebahn S., Görner K., Lindenberger D., Markewitz P., Merten F., Stolten D. (2017). Re-energizing energy supply: Electrolytically-produced hydrogen as a flexible energy storage medium and fuel for road transport. J. Power Sources.

[17] Zhao K., Fang X., Cui C., Kang S., Zheng A., Zhao Z. (2022). Co-production of syngas and H_2_ from chemical looping steam reforming of methane over anti-coking CeO_2_/La_0.9_Sr_0.1_Fe_1−x_Ni_x_O_3_ composite oxides. Fuel.

[18] Peláez-Peláez S., Colmenar-Santos A., Pérez-Molina C., Rosales A.-E., Rosales-Asensio E. (2021). Techno-economic analysis of a heat and power combination system based on hybrid photovoltaic-fuel cell systems using hydrogen as an energy vector. Energy.

[19] Karimkashi S., Kahila H., Kaario O., Larmi M., Vuorinen V. (2020). Numerical study on tri-fuel combustion: Ignition properties of hydrogen-enriched methane-diesel and methanol-diesel mixtures. Int. J. Hydrogen Energy.

[20] Wang H., Ji C., Shi C., Yang J., Ge Y., Wang S., Chang K., Meng H., Wang X. (2022). Parametric modeling and optimization of the intake and exhaust phases of a hydrogen Wankel rotary engine using parallel computing optimization platform. Fuel.

[21] Gomez A., Smith H. (2019). Liquid hydrogen fuel tanks for commercial aviation: Structural sizing and stress analysis. Aerosp. Sci. Technol..

[22] Shi C., Zhang Z., Ji C., Li X., Di L., Wu Z. (2022). Potential improvement in combustion and pollutant emissions of a hydrogen-enriched rotary engine by using novel recess configuration. Chemosphere.

[23] Collodi G. (2010). Hydrogen Production via Steam Reforming with CO_2_ Capture. Chem. Eng. Trans..

[24] Santhanam K.S., Press R.J., Miri M.J., Bailey A.V., Takacs G.A. (2017). Introduction to Hydrogen Technology.

[25] Takeda S., Nam H., Chapman A. (2022). Low-carbon energy transition with the sun and forest: Solar-driven hydrogen production from biomass. Int. J. Hydrogen Energy.

[26] Lakhlifi A., Dahoo P.R., Picaud S., Mousis O. (2015). A simple van’t Hoff law for calculating Langmuir constants in clathrate hydrates. Chem. Phys..

[27] Jiang W., Sun P., Li P., Zuo Z., Huang Y. (2021). Transient thermal behavior of multi-layer insulation coupled with vapor cooled shield used for liquid hydrogen storage tank. Energy.

[28] Nguyen D.H., Kim J.H., Vo T.T.N., Kim N., Ahn H.S. (2022). Design of portable hydrogen tank using adsorption material as storage media: An alternative to Type IV compressed tank. Appl. Energy.

[29] Su Y., Lv H., Zhou W., Zhang C. (2021). Review of the Hydrogen Permeability of the Liner Material of Type IV On-Board Hydrogen Storage Tank. World Electr. Veh. J..

[30] Molkov V., Dadashzadeh M., Kashkarov S., Makarov D. (2021). Performance of hydrogen storage tank with TPRD in an engulfing fire. Int. J. Hydrogen Energy.

[31] Ahamed M.I., Shakeel N., Anwar N., Khan A., Asiri A.M., Dzudzevic-Cancar H., Sen F., Khan A., Asiri A.M. (2021). 4—Graphene-based nanocomposite for hydrogen storage application. Micro and Nano Technologies, Nanomaterials for Hydrogen Storage Applications.

[32] Zhang L., Ren D., Ding W. (2022). High hydrogen storage ability of a decorated g-C_3_N_4_ monolayer decorated with both Mg and Li: A density functional theory (DFT) study. Int. J. Hydrogen Energy.

[33] Özdoğan E., Hüner B., Süzen Y.O., Eşiyok T., Uzgören I.N., Kıstı M., Uysal S., Selçuklu S.B., Demir N., Kaya M.F. (2022). Effects of tank heating on hydrogen release from metal hydride system in VoltaFCEV Fuel Cell Electric Vehicle. Int. J. Hydrogen Energy.

[34] Manoharan Y., Hosseini S.E., Butler B., Alzhahrani H., Senior B.T.F., Ashuri T., Krohn J. (2019). Hydrogen Fuel Cell Vehicles; Current Status and Future Prospect. Appl. Sci..

[35] Malleswararao K., Aswin N., Kumar P., Dutta P., Murthy S.S. (2022). Experiments on a novel metal hydride cartridge for hydrogen storage and low temperature thermal storage. Int. J. Hydrogen Energy.

[36] Bai X.-S., Yang W.-W., Yang Y.-J., Zhang K.-R., Yang F.-S. (2022). Multi-variable optimization of metal hydride hydrogen storage reactor with gradient porosity metal foam and evaluation of comprehensive performance. Int. J. Hydrogen Energy.

[37] Banerjee S., Mukhopadhyay P. (2007). Interstitial Ordering. Pergamon Materials Series.

[38] Matysina Z.A., Gavrylyuk N.A., Kartel M.T., Veziroglu A., Veziroglu T.N., Pomytkin A.P., Schur D.V., Ramazanov T.S., Gabdullin M.T., Zolotarenko A.D. (2021). Hydrogen sorption properties of new magnesium intermetallic compounds with MgSnCu_4_ type structure. Int. J. Hydrogen Energy.

[39] Chang S., Young K.-H., Ouchi T., Meng T., Nei J., Wu X. (2016). Studies on Incorporation of Mg in Zr-Based AB_2_ Metal Hydride Alloys. Batteries.

[40] Young K.-H., Nei J., Wan C., Denys R.V., Yartys V.A. (2017). Comparison of C14- and C15-Predomiated AB_2_ Metal Hydride Alloys for Electrochemical Applications. Batteries.

[41] Eisapour A.H., Eisapour M., Talebizadehsardari P., Walker G.S. (2021). An innovative multi-zone configuration to enhance the charging process of magnesium based metal hydride hydrogen storage tank. J. Energy Storage.

[42] Anbarasu S., Muthukumar P., Mishra S.C. (2014). Tests on LmNi_4.91_Sn_0.15_ based solid state hydrogen storage device with embedded cooling tubes—Part A: Absorption process. Int. J. Hydrogen Energy.

[43] Kunwar B., Cheng H.N., Chandrashekaran S.R., Sharma B.K. (2016). Plastics to fuel: A review. Renew. Sustain. Energy Rev..

[44] Li G., Chen Z., Li Y., Zhang D., Yang W., Liu Y., Cao L. (2020). Engineering Substrate Interaction to Improve Hydrogen Evolution Catalysis of Monolayer MoS_2_ Films beyond Pt. ACS Nano.

[45] Qu J., Li Y., Li F., Li T., Wang X., Yin Y., Ma L., Schmidt O.G., Zhu F. (2022). Direct Thermal Enhancement of Hydrogen Evolution Reaction of On-Chip Monolayer MoS_2_. ACS Nano.

[46] Tekalgne M.A., Van Nguyen K., Nguyen D.L.T., Nguyen V.-H., Nguyen T.P., Vo D.-V.N., Trinh Q.T., Hasani A., Do H.H., Lee T.H. (2020). Hierarchical molybdenum disulfide on carbon nanotube-reduced graphene oxide composite paper as efficient catalysts for hydrogen evolution reaction. J. Alloys Compd..

[47] Li Y., Wang H., Xie L., Liang Y., Hong G., Dai H. (2011). MoS_2_ Nanoparticles Grown on Graphene: An Advanced Catalyst for the Hydrogen Evolution Reaction. J. Am. Chem. Soc..

[48] Zhang R., Zhang M., Yang H., Li G., Xing S., Li M., Xu Y., Zhang Q., Hu S., Liao H. (2021). Creating Fluorine-Doped MoS_2_ Edge Electrodes with Enhanced Hydrogen Evolution Activity. Small Methods.

[49] Zhang L., Jin Z., Tsubaki N. (2022). Activating and optimizing the MoS_2_@MoO_3_ S-scheme heterojunction catalyst through interface engineering to form a sulfur-rich surface for photocatalyst hydrogen evolution. Chem. Eng. J..

[50] Liao T., Sun Z., Sun C., Dou S.X., Searles D.J. (2014). Electronic Coupling and Catalytic Effect on H_2_ Evolution of MoS_2_/Graphene Nanocatalyst. Sci. Rep..

[51] Tsai C., Li H., Park S., Park J., Han H.S., Nørskov J.K., Zheng X., Abild-Pedersen F. (2017). Electrochemical generation of sulfur vacancies in the basal plane of MoS_2_ for hydrogen evolution. Nat. Commun..

[52] Khan M., Yousaf A.B., Chen M., Wei C., Wu X., Huang N., Qi Z., Li L. (2016). Molybdenum sulfide/graphene-carbon nanotube nanocomposite material for electrocatalytic applications in hydrogen evolution reactions. Nano Res..

[53] Wang X., Dai J., Xie H., Yang C., He L., Wu T., Liu X., Xu Y., Yuan C., Dai L. (2022). In-situ construction of ultrathin MoP-MoS_2_ heterostructure on N, P and S co-doped hollow carbon spheres as nanoreactor for efficient hydrogen evolution. Chem. Eng. J..

[54] Chen J., Li S., Tao Z. (2003). Novel hydrogen storage properties of MoS_2_ nanotubes. J. Alloys Compd..

[55] Passamonti F.J., Sedran U. (2012). Recycling of waste plastics into fuels. LDPE conversion in FCC. Appl. Catal. B Environ..

[56] Al-Salem S.M., Antelava A., Constantinou A., Manos G., Dutta A. (2017). A review on thermal and catalytic pyrolysis of plastic solid waste (PSW). J. Environ. Manag..

[57] Zhang P., Liang C., Wu M., Chen X., Liu D., Ma J. (2022). High-efficient microwave plasma discharging initiated conversion of waste plastics into hydrogen and carbon nanotubes. Energy Convers. Manag..

[58] Jagodzińska K., Jönsson P.G., Yang W. (2022). Pyrolysis and in-line catalytic decomposition of excavated landfill waste to produce carbon nanotubes and hydrogen over Fe- and Ni-based catalysts—Investigation of the catalyst type and process temperature. Chem. Eng. J..

[59] Yao D., Zhang Y., Williams P.T., Yang H., Chen H. (2018). Co-production of hydrogen and carbon nanotubes from real-world waste plastics: Influence of catalyst composition and operational parameters. Appl. Catal. B Environ..

[60] Assefi M., Mofarah S.S., Maroufi S., Nekouei R.K., Wang W., Kert E., Sahajwalla V. (2022). Regeneration of hydrogen through thermal micronisation of end-of-life polymers for sustainable reduction of iron oxide. Fuel Process. Technol..

[61] Zhang Y., Huang J., Williams P.T. (2017). Fe–Ni–MCM-41 Catalysts for Hydrogen-Rich Syngas Production from Waste Plastics by Pyrolysis–Catalytic Steam Reforming. Energy Fuels.

[62] Liu X., Zhang Y., Nahil M.A., Williams P.T., Wu C. (2017). Development of Ni- and Fe-based catalysts with different metal particle sizes for the production of carbon nanotubes and hydrogen from thermo-chemical conversion of waste plastics. J. Anal. Appl. Pyrolysis.

[63] Wu C., Williams P.T. (2014). Hydrogen from waste plastics by way of pyrolysis–gasification. Proc. Inst. Civ. Eng.-Waste Resour. Manag..

[64] Aminu I., Nahil M.A., Williams P.T. (2022). High-yield hydrogen from thermal processing of waste plastics. Proc. Inst. Civ. Eng.-Waste Resour. Manag..

[65] Barthelemy H., Weber M., Barbier F. (2017). Hydrogen storage: Recent improvements and industrial perspectives. Int. J. Hydrogen Energy.

[66] Yusaf T., Fernandes L., Abu Talib A.R., Altarazi Y.S., Alrefae W., Kadirgama K., Ramasamy D., Jayasuriya A., Brown G., Mamat R. (2022). Sustainable Aviation—Hydrogen Is the Future. Sustainability.

[67] Razi F., Dincer I. (2022). Challenges, opportunities and future directions in hydrogen sector development in Canada. Int. J. Hydrogen Energy.

